# Pedagogical innovation and educator wellbeing in UK audiology teaching: cross-institutional insights from the COVID-19 experience

**DOI:** 10.3389/fmed.2025.1597240

**Published:** 2025-10-01

**Authors:** Srikanth Chundu, Natalie Morley, Cathrine V. Jansson-Boyd, Bhavisha Parmar

**Affiliations:** ^1^School of Psychology, Sports and Sensory Sciences, Anglia Ruskin University, Cambridge, United Kingdom; ^2^Faculty of Brain Sciences, Ear Institute, University College London, London, United Kingdom; ^3^SOUND Lab, Department of Clinical Neurosciences, University of Cambridge, Cambridge, United Kingdom

**Keywords:** pedagogy, audiology, teaching, higher education, health education

## Abstract

**Background:**

Academic teaching staff in higher education routinely balance multiple roles, including teaching, research, and pastoral student care. The onset of the COVID-19 pandemic, which forced the closure of university campuses, significantly intensified these demands. To maintain the continuity and quality of education, staff were required to swiftly adapt and implement new, robust teaching methods. This sudden shift placed additional pressure on an already stretched workforce. This study aims to investigate the impact of the COVID-19 pandemic on audiology education in the United Kingdom (UK), with a particular focus on the work-related wellbeing of academic teaching staff.

**Methods:**

A qualitative study was conducted using semi-structured interviews with eleven teaching staff involved in audiology higher education across the UK.

**Results:**

The following four themes were identified (1) initial institutional response to the pandemic, (2) adapting practical audiology training through teleaudiology and patient simulation, (3) supporting students whilst safeguarding staff wellbeing, and (4) pedagogical approaches for a unique, technology-driven audiology profession.

**Conclusion:**

The detrimental impact of the pandemic on academic staff well-being was evident in the form of increased workloads and escalating institutional pressures, which frequently prioritized student well-being over that of staff. Audiology HEI educators acknowledged that while audiology training cannot be entirely conducted online due to the essential hands-on skills that must be practiced in person, a hybrid or blended learning approach could be beneficial.

## 1 Introduction

During the national lockdowns prompted by the COVID-19 pandemic, higher education institutions (HEIs), around the world, had to adapt teaching techniques. They either had to be fully deliverable online or structured as blended learning/hybrid modes of delivery. Consequently, lecturers and teaching staff reassessed their approaches and developed new strategies for teaching delivery. This involved substantial and disruptive challenges for the majority of the world’s higher education providers (1).

Prior to the pandemic, HEIs across the UK were already incorporating online technologies to some extent, utilizing tools such as streaming, website sharing, simulation programs, and online learning environments to enrich their content (2, 3). The use of digital technologies to deliver online teaching has been advantageous in enhancing inclusive education, and creating flexible, convenient workspaces (4, 5). However, reliance on online education delivery has been known to have challenges including the need for effective, consistent technological infrastructure, sufficient student- instructor and student-student interaction and engagement, and instructor confidence to employ online teaching techniques (6).

For medical courses, students need practical and clinical experience to complement their theoretical knowledge. They would also need practical assessments before gaining healthcare accredited qualifications. A pre-pandemic review identified several barriers to the implementation of online learning in medical higher education, including time constraints, insufficient technical skills among educators, and inadequate infrastructure (7). Stoehr et al. (8) described the COVID-19 pandemic as a catalyst for a new “online era” in medical education. However, a specific concern for some students was the lack of practical training when teaching transitioned to online during the pandemic (9).

Research from Turkey investigating audiology students opinions during the pandemic found that 41.8% of students felt the national lockdown limited clinical practices, and 67% felt that online courses did not contribute to their professional healthcare competencies (10). A questionnaire-based study examining the perceptions of Israeli students and preceptors during practical audiology training pre and post pandemic found that the pace at which the clinical competencies were taught was significantly faster during the pandemic but students who took practical assessments before the pandemic felt more prepared and confident in theoretical knowledge compared to those assessed during the pandemic. The primary adverse impact of the pandemic was identified as a reduction in clinical hours dedicated to the development of practical clinical skills (11). These findings are consistent with existing literature that indicates online audiology education may limit active participation and may inadequately represent the application of theoretical concepts in practical settings (12). A systematic review on the use of simulation in audiology education around the world suggested there is a lack of high quality literature in this area and the use of simulated clinical practice in audiology learning seemed to be less effective compared to other health professions (13). However, Svec et al. (14) described the available audiology simulation tools commonly used by educators and reported that the majority of surveyed educators in the United States felt that virtual audiology education tools were easy to use, and improved teaching and learning (14). The use of telemedicine tools for audiology training also has significant advantages for low resource areas (15).

Research suggests that initiatives aimed at enhancing student well-being can inadvertently have a negative impact on staff wellbeing due to increased demands and workload (16, 17). In a study conducted by Miguel et al. (18) on mental health and remote teaching challenges faced by medical lecturers, around 42% of the lecturers reported personal burnout and 16% reported student related burnout. The burnout included changes to their sleep routines, and increased stress (18). Furthermore, Mahlaba and Mentz (19) reported both positive and negative experiences faced by the lecturers due to the pandemic, as lecturers who had an experience with online teaching faced less challenges (19).

Despite the extensive literature examining students’ perspectives and challenges in learning during the pandemic, there remains a notable gap in research regarding the experiences of HEI educators from a cross institutional perspective and disciplines (20). Therefore, the aim of this study is to investigate how audiology educators in UK HEIs adapted their teaching methods during COVID-19 pandemic and the subsequent effects on their wellbeing by using semi structured interviews. Previous studies have used questionnaires to collect data in this area; however, the present study uses semi structured interviews to allow for greater flexibility and depth. This method facilitates the collection of rich and nuanced data by creating a conversational atmosphere, which helps to put participants at ease and encourages the sharing of deeper insights (21).

## 2 Materials and methods

### 2.1 Ethical consideration

Ethical approval was obtained from faculty of science and engineering ethics panel, Anglia Ruskin University, Cambridge, UK (Reference number: FREP/SREP: 0621-02).

### 2.2 Design and participants

All participants were academics working in Audiology education within UK universities at the onset of the COVID-19 pandemic. Participants were in various stages of their careers. At the time of conducting this research, there were 14 universities offering audiology programs in the UK. Participants were invited to engage in semi-structured interviews conducted one on one via the online platform Microsoft Teams, where they were asked to share their experiences of teaching during the pandemic. Participants were required to confirm that they were over the age of 18 and currently employed as audiology educators at a UK university. A total of 11 participants were recruited from eight different providers of audiology higher education courses across the UK. The participants had an average of 10 years of work experience (SD = 4.7), with varying levels of experience in online teaching. Five participants had 1 year or less of experience in online delivery.

### 2.3 Recruitment strategy

Participants were recruited through purposive sampling, a non-random technique that selects individuals based on specific characteristics, expertise, or experiences directly relevant to the research (22). The study was advertised through social media (X (formerly twitter) and mailing lists and academic online channels. The advert invited participants who were teaching audiology programs within UK universities to take part in an online interview discussing how the COVID-19 pandemic affected audiology teaching practices. Upon receiving an inquiry via email, participants were sent a participant information sheet, and a consent form. Once the consent form was completed and returned, the interview time and date were scheduled.

### 2.4 Interviews

Interview questions were developed by two researchers (SC and CJB). The questions were discussed with another researcher (NM). A guide was created for the interviewer to help deliver the interviews, this made sure that the questions were asked in the same order every time and that they were delivered in a uniform and coherent way. Trial runs were conducted using the guide, to ensure that questions were asked by the interviewer in a consistent and neutral manner to avoid leading or influencing participant responses CJB and NM). The interviews were conducted by NM via Microsoft Teams and lasted approximately 1 h. Prior to initiating the recording, participants were asked to reconfirm their consent for both video and audio recording. After consent was obtained, the researcher (NM) introduced themselves, provided an overview of the study’s purpose, and explained the ethical considerations involved. Once the participants confirmed their understanding and agreement, the interview proceeded. Interviews took place between August and September 2021.

### 2.5 Data analysis

Reflexive inductive thematic analysis (23, 24) was carried out to analyze the transcripts (SC, NM, BP). The research team followed the recommended step-by-step thematic analysis method (24), led by BP, an audiology educator with prior experience in this method. The process comprised the following stages: familiarization with the data, coding, generation of initial themes, review of themes, definition and naming of themes, and final write-up. This process involved iterative coding, theme development, and reflection to identify patterns of shared meaning. Coding was collaborative, and differences in coding terminology was discussed between researchers to generate ideas and construct the final code list. Initial codes were iteratively reviewed and refined into broader, synthesized themes through sustained engagement with the data. Theme interpretations were discussed, reviewed, and refined to enhance clarity and ensure a coherent and logically structured narrative (BP, SC).

## 3 Results

Four themes were identified demonstrating the impact of the pandemic on audiology pedagogy and educator well being (see Table 1).

**TABLE 1:** Theme overview.

Theme	Theme description
1 Initial institutional response to the pandemic	This theme outlines how higher education institutes responded to the COVID-19 pandemic and the process of information relay to teaching departments to inform changes in teaching process
2 Adapting practical audiology training through teleaudiology and patient simulation	This theme underscores the central role of practical training in audiology education and illustrates how innovative approaches were employed to replace traditional patient-facing instruction during the pandemic.
3 Supporting students whilst safeguarding staff well-being	This theme explores the impact of workload and work pattern changes on audiology educators during the pandemic.
4 Pedagogical approaches for a unique, technology-driven audiology profession	This theme captures insights from teaching during the pandemic and identifies pedagogical approaches that could be integrated into future practice.

### Theme 1: Initial institutional response to the pandemic

Participants described how their teaching institutes responded to the national restrictions following the outbreak of COVID-19. This included specific guidance that was created and how that information was relayed to staff members.

*“and there’s no breathing space for us. it’s hard. It is, interesting because very little was explicitly said about what we needed to do. but ultimately you just get on with it”*

*“large scale decisions were made by the top echelons of the university and handed over to local staff to implement with absolutely no guidance for how that would work in their departments so students would be told this is happening you know we’re going online”*

*“we were expected to just all of a sudden perform online in a completely different format and on paper it felt that all the guidance was there but in practice I didn’t really know how to do things we like there wasn’t as much guidance”*

*“it was pretty chaotic because the underpinning structures were not there.”*

Participants used their previous teaching experience to implement the best possible teaching solutions during the pandemic but broadly felt unprepared. There was a stated lack of explicit guidance as to what they needed to do and thus based on previous teaching experiences they re-evaluated what they did to adjust or find new ways to implement teaching. Audiology teachers also expressed how different levels of experience of using online teaching methods impacted the initial response to the pandemic.

*“you know they put out papers on like pivoting to online education and blended learning. Again, we probably haven’t had quite so much struggle as other course simply because we’ve been teaching like that for years”*

*“the team had well about 10 years of experience of doing the other courses online or blended learning it wasn’t an issue so much to move to online for the theory”*

### Theme 2: Adapting practical audiology training through teleaudiology and patient simulation

Audiology is a healthcare field that requires practical, hands-on training. Participants highlighted the challenges associated with delivering both instructional and clinical components remotely, noting that remote clinical audiology was particularly uncommon prior to the pandemic.

**“***it’s very disconcerting to our discipline because it’s a practical discipline and you kind of have to do things face to face or at the time*… *clinics weren’t online before COVID”*

*“I think one of the problems is practical aspects of our programs are very, very difficult to deliver remotely. So, we tried to work around that.”*

Audiology educators highlighted the significance of practical teaching components in audiology education. Some educators reported that online patient simulation software was beneficial for allowing students to practice clinical skills, such as pure tone audiometry. Others utilized videos of clinical scenarios to facilitate teaching and assessment.

*“we’ve had to go to patient simulation because we haven’t been able to get the patient volunteers because of the age profile of the patients they’re all sort of elderly and retired people who tend to wear hearing aids they tend to be the bread and butter of audiology and obviously for a while before the vaccination program started. they were high risk* [for contracting the virus]”

*“For some of the procedures where they may be more straightforward, we allowed them to do it in their own clinic and upload the video and we assessed the video, but some of those procedures don’t lend themselves well to being videoed.”*

*“The university invested in software so we have some which allows us to do recorded lectures with captions and transcripts and for us in particular they invested in software that allows you to do pure tone audiometry remotely”*

When students were able to practice clinical skills on site, social distancing guidance restricted the numbers of staff and students that were able to use the teaching lab facilities at any one time. Personal, protective equipment (PPE) was also required during the on-site training, but some participants reported the use of masks to have an impact on overall communication between teacher and student.

*“Our clinic rooms are quite small* [and they] *only allow for limited occupation of those rooms for safety reasons. We’ve had to split the skills delivery into small groups so we have half the students coming in the morning and then the other half come in in the afternoon so they’re not getting they usually get a full day together and when the campus was shut. We ended up taking equipment and demonstrating on fake ears or fake heads as best we could”*

*“it was definitely an adjustment to wearing PPE and being a hearing aid wearer myself I really struggled to hear and the students actually say that they struggle to hear us as well and understand us especially if English is their second language so it was more noticeable with that as well”*

Some participants reported the barrier of poor internet connection while using live online video conferencing to teach.

*“I found for me that worked better than trying to teach them live because their internet connections are bad, that that was the other issue”*

### Theme 3: Supporting students whilst safeguarding staff wellbeing

Audiology teachers discussed the additional pressure on work-life balance when teaching during the pandemic. Specifically, participants mentioned how they wanted to ensure the student’s experience of higher education, and the quality of teaching was the same as pre-pandemic.

*“I have to answer emails and the only time to answer the emails is after work so before it was hard enough to separate home and work life having all of this at home.”*

*“There is that pressure to continue delivering higher education even when everything else has stopped you know even when students aren’t at work and aren’t with people. It was the expectation that the courses would continue to run”*

*“The students are my priority and they should be everybody’s priority so. I constantly felt bad because I didn’t truly feel that they were getting the experience that they were paying for”*

*“This year we’ve been doing resits exam boards first assessments and clinical assessments and that’s still going and our next semester starts next week and we haven’t finished. So, getting to take annual leave has been almost impossible. You know even though you’ve been on annual leave you haven’t been able to switch your email off and go away because it just hasn’t been able to happen”*

Participants faced significant challenges in using their personal time to dedicate to student well-being. The need to ensure that students receive adequate support may have exacerbated an already complex situation. This is evident in the efforts to maintain a student-centered approach, as audiology lecturers worked beyond typical hours to support students during a highly distressing period.

*“Well it really it was to try and give the students as good an experience as possible try not to disadvantage them as much as possible and just generally try and help the students through this distressing time”*

*“It’s hard. It is hard because you’re really, really trying to do your best for people because this is their jobs their livelihoods. Most people on my courses are more mature with kids with mortgages with bills to pay and they’re saying to you if I don’t get through this I might lose my job and you’re doing everything in your power to make sure that that they can- but that does take a toll.”*

*“When you’ve been furloughed and people have been made redundant you know people didn’t want to end up in that situation so there has there has been some pressure we do have students in audiology who needed their transcript to be able to start* [work] *in September so we had to get them through as well otherwise they’d have had to wait a year.”*

### Theme 4: Pedagogical approaches for a unique, technology-driven audiology profession

Audiology education occupies a distinct space within the health sciences, shaped by its reliance on advanced diagnostic technologies, specialized equipment, and a combination of technical expertise and patient interaction. Audiology teachers reported certain benefits of using a certain amount of online teaching for audiology higher education, beyond the pandemic. These included flexibility for part time students, more effective use of technology and digital tools, and the inclusion of a more diverse range of speakers/teaching staff from around the world.

**“***yeah I think there is definitely some room for what they call a hybrid or blended approach as they don’t need to be there* [on campus] *like 100% of your time. But I personally don’t think it can be 100% online either”*

*“it’s a lot more flexible so like part-time students definitely benefited from it and you can attend short courses so people from all over the world can attend and also can speak at your program so it made it a lot more open”*

*“we’ve been forced to do remotely which we have always probably said in the past we can’t do that and now I think well yeah you can do that if you if you think about how you mitigate for certain things so maybe it can be a little more flexible”*

Communication between students and staff has also changed as meetings have been held virtually. Participants reported this to be a more flexible approach.

*“I’ve actually found communication with students better since we’ve been online because we’ve met via teams then sometimes when they’re in university and then you say “oh pop in” and we’ll have a personal tutorial and they forget. Whereas using teams you know we will continue to use that. Especially* [for] *students who are on placement- it’s been so much better”*

*“It’s more about the supporting*… *I’ve always been in regular contact with students that I’m personal tutor for and students in placement, but actually I’ve learned something positive from it about using the technology to have even better communication with students and I think that’s been a really positive learning thing.”*

Finally, audiology teaching needs to remain in tune with primary healthcare as service delivery models have changed in the pandemic.

*“I think we will need to maybe adapt the teaching to the changes in audiology delivery at primary care level because during the pandemic how they delivered services changed*…*they were doing telehealth and taking history over the phone, so*…*if those some of those changes stay in place for the future because clinics find they can run them more successfully we might have to adapt our teaching but within audiology we’re adapting all the time because the technology changes all the time so there may be changes because of COVID.”*

## 4 Discussion

This study is one of the few that has investigated the impact of COVID-19 pandemic on audiology teaching across UK HEIs. Participants in the present study reported that changes to work methodologies and work patterns during the pandemic had a negative impact on their work-life balance. This aligns with existing literature examining the merging of higher education teaching and home environments during this period, which often resulted in educators experiencing heightened stress and feelings of overwhelm (burnout) due to the integration of their professional teaching spaces into personal home settings (25). There was an implicit expectation that educators should be continuously available to students to enhance student experience. However, evidence indicates that constant availability can have detrimental effects on well-being, as the inability to disengage mentally and physically from work negatively impacts both personal health and professional effectiveness, ultimately affecting the quality of teaching and learning outcomes (26). Moreover, although academics play a critical role in providing pastoral support to students, the impact of these responsibilities on educator well-being is frequently underrecognized in the literature. (27). A study examining the mental well-being of UK academics from May to September 2020 reported significantly low levels of well-being, with scores falling below pre-pandemic baseline levels observed in the general population (28).

During the pandemic, there was a reduction in the number of students that could be in the labs due to social distancing rules and there was an increased use of PPE during practical teaching. The assessment of practical competencies was adapted in accordance with pandemic-related restrictions, ensuring continued alignment with national accreditation standards. Although participants felt they had to amend the curriculum to deliver in the best possible way, they were still unsure if the students were getting the clinical experience they would need. In a study by Brand et al. (11), audiology supervisors reported no difference in theoretical training or supervision quality between pre- and during-pandemic students, though practicals were introduced more gradually before the pandemic. Pre-pandemic students noted receiving more theory, a steadier progression of competencies, and greater placement variety. Both groups reported comparable preparedness for clinical work, highlighting the importance of supervisors strategically prioritizing core training components to ensure graduate employment readiness.

Participants noticed a lack of infrastructure required to implement online learning and support students and staff during a pandemic. Nandy et al. (29) reported that although pandemics are a very rare occurrence, this scenario has uncovered gaps in the current HEI teaching models (30) that are focused on person-environment fit model (31) referring to the compatibility between a person and their environment/organization. Findings from this study further illustrate that, although audiology HEI educators often demonstrated resilience and motivation to explore innovative teaching methods, the swift and substantial shifts necessitated by the national lockdown posed considerable challenges and strain. This is similar to further studies where academics faces challenges such as frustration and stress in adapting their teaching swiftly in response to pandemic (32). Overall, the use of virtual clinical simulation tools for audiology HEIs was positive, and there was an acknowledged importance of implementing these as teleaudiology becomes more common practice. Confidence in using online or blended learning tools is influenced by educators’ experience and the availability of appropriate infrastructure. A qualitative study of students and educators exploring medical education during the pandemic found a lack of standardization of e-learning tools, and the need for dedicated teams to support and troubleshoot technical difficulties (33). There is currently no consensus on the type and quality of audiology simulation software that should be used for audiology HEI education. Further guidance would support educators and help to standardize processes. Students accessing such simulation software in their personal study time would also increase their skills practice, rather than relying solely on clinical placement with supervision.

Although the pandemic has ended, it remains essential for academics and universities to reflect on the lessons learned to enable more agile responses to future disruptions. These insights also offer valuable opportunities to enhance accessibility and ensure the continued delivery of high-quality education. Nandy et al. (29) summarized key recommendations that have stemmed from HEI teaching during the pandemic. These range from the need to audit, monitor and evaluate current teaching practices to uncover strengths and weaknesses, explore opportunities and collaborations, establish long term measures to strengthen resilience in the HEI workforce and prioritize the balance between student and educator well-being (30). Furthermore, universities and academic staff invested significant time and resources in adapting teaching methods and acquiring new skills in response to the pandemic. In a technology-driven field such as audiology, it is important to build on the positive teaching experiences gained during this period to develop more inclusive and forward-looking pedagogical approaches, rather than reverting entirely to pre-pandemic models (34).

### 4.1 Strengths and limitations

A principal strength of this study lies in its extensive cross-institutional representation, encompassing perspectives from over 50% of higher education institutions that offer audiology programs in the UK. Given the relatively small and highly specialized nature of the audiology field, this level of coverage is particularly significant. To date, this is the only known study to explore the experiences of audiology teaching staff during the COVID-19 pandemic across multiple UK institutions. As such, it provides a valuable account of the varied institutional responses within a single national context, highlighting the heterogeneity of approaches adopted across the sector. Additionally, the timing of the interviews–conducted shortly after the lifting of national lockdown restrictions–provides a unique advantage. This proximity to the lockdown period likely improved the accuracy and detail of participants’ reflections on that challenging time, as their recall was still fresh, offering valuable insights into the immediate impacts of the pandemic on audiology education. However, as participants were reflecting on their experiences shortly after the lockdowns, their perspectives may be influenced by the emotional and operational stresses of the period. This immediacy may also mean that longer-term impacts and adjustments made post-lockdown are not fully captured, limiting insights into more sustainable changes that could influence audiology education in the future.

### 4.2 Future considerations

Future research should track how educational provision for healthcare subjects, including audiology, has evolved since the pandemic, with a particular focus on the integration of online and hybrid tools into course curricula. Understanding the long-term adaptations in healthcare education will provide valuable insights into the sustainability and effectiveness of these changes, especially as digital tools are applied not only to theoretical learning but also to the development of clinical skills. In shaping future educational methods, it is essential to involve a broader range of stakeholders, including not only educators and students but also patients and the public. By incorporating these additional perspectives, healthcare education can better align with the real-world needs of those it ultimately serves. Co-producing educational approaches with input from teachers, students, patients, and the public can facilitate a holistic approach to healthcare learning that integrates theoretical knowledge, clinical practice, and patient-centered care. This collaborative approach would ensure that digital technologies and hybrid learning tools are designed and implemented in ways that benefit all stakeholders, enhancing both learning outcomes and patient care. By actively engaging educators, students, patients, and the public in co-designing educational strategies, healthcare programs can create more inclusive, innovative, and practical learning environments that foster both academic knowledge and essential clinical competencies. Furthermore, it could lead to the development of educational models that are both technologically advanced and responsive to the evolving needs of future clinicians and the communities they serve.

## 5 Conclusion

This study explored the perceptions of audiology academics across UK universities regarding the impact of the COVID-19 pandemic on teaching practices and academic wellbeing. The findings demonstrate that, regardless of institutional affiliation, educators encountered similar challenges and responses during this period. The pandemic prompted a rapid and unprecedented shift in the delivery of audiology education, with educators having to quickly adapt to online and blended teaching approaches. Those with prior experience in remote teaching transitioned more easily, while others faced a steep learning curve in the absence of clear institutional guidance. Participants consistently reported increased workloads, heightened student support demands, and blurred boundaries between work and personal time, all of which negatively affected their wellbeing. Despite these difficulties, the study also uncovered positive developments that could help shape the future of audiology education. Educators recognized that while core hands-on clinical training must remain in person, blended learning models offer valuable flexibility and accessibility. Moreover, remote teaching methods align well with the growing use of telehealth in healthcare delivery, providing students with relevant, transferable skills. By capturing the lived experiences of audiology educators during the pandemic, this study contributes to a deeper understanding of how higher education responded to crisis conditions. It also highlights opportunities for sustainable, flexible teaching models that support both student learning and staff wellbeing. These findings can inform future curriculum planning, institutional policy, and staff development initiatives across health education disciplines

## Data Availability

The raw data supporting the conclusions of this article will be made available by the authors, without undue reservation. Further inquiries can be directed to the corresponding author.
